# The psychiatric assessment including fitness to participate in court proceedings for defendants with intellectual disabilities

**DOI:** 10.1192/j.eurpsy.2024.105

**Published:** 2024-08-27

**Authors:** J. McCarthy

**Affiliations:** King’s College London, London, United Kingdom

## Abstract

**Abstract:**

The very brief presentation will describe the initial approach to the psychiatric assessment of the case presented including the issue of fitness to participate in court proceedings. The use of screening tools at court to identify people with intellectual disabilities will be highlighted along with what support can be provided for vulnerable offenders in attending court. The potential disposal options for the court such as prison or hospital or community will be outlined. Variation in practice across Eurpean countries needs further discussion to ensure the rights of defendants with intellectual disabilities are safeguarded during their participation in court proceedings

**Disclosure of Interest:**

None Declared

